# Safety of local anesthetics^☆^^☆☆^

**DOI:** 10.1016/j.abd.2019.09.025

**Published:** 2019-12-18

**Authors:** Ana Carolina Figueiredo Pereira Cherobin, Glaysson Tassara Tavares

**Affiliations:** Dermatology Service, Hospital das Clínicas, Universidade Federal de Minas Gerais, Belo Horizonte, MG, Brazil

**Keywords:** Anaphylaxis, Anesthetics, local, Drug-related side effects and adverse reactions

## Abstract

Local anesthetics are essential medications for the conduction of dermatological procedures. They stop the depolarization of nerve fibers and are divided into two main categories, the amide and ester types. Systemic toxicity with reflex on the central nervous and cardiovascular systems is their most feared adverse reactions, and the anaphylactic reaction is the most concerning one. Although potentially fatal, these events are extremely rare, so local anesthetics are considered safe for use in in-office procedures.

## Introduction

In-office dermatological procedures are usual and include from the biopsy of minor injuries to cosmiatric procedures and long complex surgeries. Local anesthesia is essential for the viability of, and good tolerance to, their conduction. This method is safe and recommended for most procedures performed outside the out- and inpatient environment.^1^, ^2^ The aim of the present study was to make a literature review about the safety of applying local anesthetics with emphasis on adverse reactions.

## Methods

The methodology used to select the articles was based on the PICO method (Problem/Patient/Population, Intervention/Indicator, Comparison and Outcome). Patients subjected to dermatological procedures composed the population of the present study, local anesthetics were the intervention and the adverse reactions were the outcomes – the comparison group was not determined.

## Results and Discussion

Results in this review were divided into the following subtitles: Action mechanisms of local anesthetics, Classification and pharmacokinetics, Types of local anesthetics, Adverse reactions, Application techniques, Safety of anesthetics and Special situations.

## Action mechanism of local anesthetics

Pain sensation depends on the ability of the nervous system to transmit electrical impulses. This propagation occurs due to different electrolyte concentrations between the intracellular region – which presents high potassium concentration (K^+^) and lower sodium concentrations (Na^+^) – and the extracellular region, where concentrations are reversed. This ionic gradient is kept by the sodium-potassium adenosine triphosphatase (Na^+^ K^+^ ATPase) pump. The external membrane of the nerve (at rest) presents positive load in comparison to the internal region due to the low membrane permeability to Na^+^ and to the action of the pump, which excludes three Na^+^ ions for each of the two internalized K^+^ ions.^3^, ^4^

The membrane becomes permeable to the Na^+^ accumulated inside the cell whenever there is stimulus over the nerve, and this process ends up in depolarization. This change in Na^+^ permeability also changes the electrical potential through the membrane; the propagation of this electrical potential is called action potential. The nerve returns to the resting state by changing the membrane permeability to Na^+^ again.^3^, ^4^, ^5^

Local anesthetics act in the Na^+^ K^+^ ATPase pump, and this process stops the sodium inflow and the propagation of pain stimulus through nervous fibers; thus, it avoids depolarization.^2^, ^5^

Pain sensation is spread through unmyelinated fibers (Fibers C), which are more sensitive to local anesthetics than myelinated fibers (Fiber A and B). This process allows sensations such as vibration and pressure to remain even after complete pain inhibition.^2^, ^5^

## Classification and pharmacokinetics

Local anesthetics are composed of a structure divided into three parts: aromatic group (lipophilic), intermediate chain and amine group (hydrophilic).

The aromatic ring provides lipid solubility to the substance; the higher the solubility, the greater the anesthetic diffusion in the nervous membrane. This property is correlated to the power of the medication.

The terminal amine has the tertiary (liposoluble) and quaternary forms (water soluble). The anesthetic is administered in its quaternary form, and its action occurs according to the proportion of molecules that turn into the tertiary form after getting in contact with the physiological pH (7.4). The ionization constant (pKa) of the anesthetic represents the pH, and half of the molecules are found in their tertiary form, and the other half is in the quaternary form. Most anesthetics have pKa similar to the physiological pH (7.4). The acidified environment favors the quaternary form whenever there is inflammation; it reduces the amount of anesthetic capable of penetrating the nerves.^5^

The intermediate chain can be composed of ester and amide, which are responsible for the anesthetic classification (Table 1).^5^, ^6^

**Table 1:** Characteristics of the most used anesthetics in dermatology.

Anesthetic	Protein affinity	Onset of action	Duration without epinephrine (min)	Duration with epinephrine (min)
*Amide type*				
Lidocaine	64	2–3 min	30–120	60–400
Mepivacaine	77	3–20 min	30–120	60–400
Bupivacaine	95	5–8 min	120–240	240–480

*Ester type*				
Procaine	5.8	5 min	15–30	30–90

Amide-type anesthetics are metabolized by the liver and they must be used carefully in patients with kidney issues. Ester-type anesthetics are degraded by plasma pseudocholinesterase and their metabolites are excreted through urine. The Para-Aminobenzoic Acid (PABA) is one of their metabolites, and it is responsible for the risk of developing allergic reactions to this group of medications. Different from what happens with anesthetics in the amide group, the potential for cross reactions between anesthetics in this group is known.^3^, ^5^, ^6^

The affinity of anesthetics with plasma proteins is correlated to the affinity of such proteins with the transmembrane sodium channels; the greater the affinity, the longer the action time of the anesthetic and its duration (Table 1).^5^, ^6^

## Types of local anesthetics

There are different methods to induce local anesthesia: topical, infiltrative, field block, peripheral nerve block and tumescent anesthesia. All these methods have transient regional anesthesia.^3^, ^5^

Substances in topical anesthesia are administered straight into the skin or into the mucosa through moisturizers, ointments, gels or sprays. Accordingly, the agent penetrates and reaches the papillary dermis to act in the endings of nerve branches.^3^, ^5^ The composite easily crosses the skin when the pka of the anesthetic gets close to the skin pH (5.5) and the corneal layer is thin (such as in the eyelids) or absent (mucosa).^7^, ^8^

Infiltrative anesthesia is obtained through medication administration right on the dermis, or through subcutaneous administration, which causes direct inhibition of nerve endings. Action starts right when the substance is injected in the dermis; however, the injection is quite painful. Pain is milder when the substance is injected in the subcutaneous tissue, but the duration time can be shorter due to the absorption of higher amounts of substance.^3^, ^5^

Field blocking consists in the deposition of anesthetic solution around the desired site (circumference shape) to anesthetize the superficial and deep nerves responsible for the innervations of the target region. Thus, it is possible anesthetizing with less product when there is no distortion in the area to be treated.^3^, ^5^

In order to block the peripheral nerve, the anesthetic must be injected around the main nerve. This method allows expressive reduction in the necessary volume of anesthetic for the procedure and prevents distortions in the operative site. These methods are broadly used in face, dactyl and nail dermatological procedures, besides demanding detailed knowledge about anatomy and acknowledged professional expertise.^3^, ^5^

The tumescent anesthesia is similar to the infiltrative one; however, it demands more diluted doses of the substance right in the region to be subjected to the procedure. Due to the lower concentration of anesthetic, one can use a larger volume of it with low toxicity risk. This technique is applied to liposuction, dermabrasion and hair implant.^5^, ^9^

### Injectable anesthetics

#### Lidocaine

Lidocaine is a systemic antiarrhythmic; nowadays, it is the most used injectable anesthetic in dermatological practices. Its action starts fast (<1 min) and its duration is intermediate (30–120 min). The maximum dose of lidocaine allowed for local infiltration in adults is 4.5 mg/kg/dose (maximum: 300–350 mg) without epinephrine and 7 mg/kg/dose (maximum: 300–500 mg) with epinephrine. The maximum dose for children under 12 years ranges from 1.5 to 2.0 mg/kg/dose (maximum: 150 mg) without epinephrine and from 3 to 4.5 mg/kg/dose (maximum: 150 mg) with it. Children over 12 years are treated with doses similar to those used for adults. This is a Category B medication during pregnancy and its kidney metabolism happens through cytochrome P450 (Table 1, Table 2).^1^, ^3^, ^6^, ^9^

#### Mepivacaine

Mepivacaine is an amide-type anesthetic that has fast action start and 30–120 min duration. Its allowed maximum dose is 300 mg with epinephrine and 500 mg without it. The dose for children is 4–6 mg/kg/dose (maximum: 270 mg) without epinephrine. Similar to lidocaine, mepivacaine is metabolized by the liver. It is a Category C in pregnancy (Table 1, Table 2).^3^, ^5^, ^6^, ^10^

#### Bupivacaine

Bupivacaine is also an amide-group anesthetic whose action start is slower than that of lidocaine (5–8 min) and its duration is longer than lidocaine's (2–4 h). Its safety for the pediatric population remains poorly established. The maximum dose in adults is 2 mg/kg/dose (175 mg in a single dose) or 400 mg/24 h without epinephrine – when epinephrine is used, it is possible using doses up to 225 mg. Its use is beneficial for prolonged procedures. Bupivacaine has cardiotoxic potential; therefore, it is necessary being cautious with patients using β-blockers or digoxin. It is a Category C in pregnancy (Table 1, Table 2).^3^, ^6^, ^10^, ^11^

#### Etidocaine

It is also a member of the amide group; its action starts between 3 and 5 min, and its duration lasts 200 min. The maximum dose recommended is 4.5 mg/kg/dose (300 mg) without epinephrine and 6.5 mg/kg/dose (400 mg) with it (Table 1).^1^

#### Prilocaine

Prilocaine belongs to the amide group and its action starts between 5 and 6 min. Its duration is intermediary, which varies from 30 to120 min. Its maximum allowable dose is 5.7–8.5 mg/kg/dose (maximum of 400 mg) without epinephrine and 600 mg with it. Its metabolism is hepatic and renal. If doses are higher than 8 mg/kg there must be risk of methemoglobinemia due to its metabolite ortho-toluidine.^1^, ^3^, ^6^

#### Ropivacaine

Ropivacaine is an amide-type anesthetic that presents slow action start (1–5 min) and long duration (2–6 h). The allowed maximum dose is 2.9 mg/kg/dose without epinephrine addition (maximum: 200 mg). Its safety in children was not confirmed so far. Similar to the other group of anesthetics, ropivacaine is metabolized by the liver. It has vasoconstrictor action and is classified in Category B in pregnancy (Table 2).^6^, ^12^

**Table 2:** Addressed doses and pregnancy categories of the mostly used local anesthetics in dermatology.

Anesthetic	Adult dosing (without epinephrine)	Adult dosing (with epinephrine)	Pediatric dosing (without epinephrine)	Pediatric dosing (with epinephrine)	Category in pregnancy
*Amide type*					
Lidocaine	4.5 mg/kg (max: 300–350)	<7 mg/kg (300–500)	4.5 mg/kg (300)	<12 years old: 4.5 mg/kg (100–150)	B
				>12 years old: <7 mg/kg (300–500)	
Mepivacaine	<300 mg	<500 mg	4–6 mg/kg (270)		C
Bupivacaine	2 mg/kg or 175 mg (single dose) or 400 mg/24 h (total dose)	225 mg	Safety and efficacy have not been established	Safety and efficacy have not been established	C
Ropivacaine	2.9 mg/kg (200 mg)		Safety and efficacy have not been established	Safety and efficacy have not been established	B

*Ester type*					
Procaine	350–500 mg	600 mg	<15 mg/kg		C

#### Articaine

Anesthetic belonging to the amide group that presents fast action start (2–4 min) and duration similar to that of lidocaine (30–120 min). Its maximum dose is 7 mg/kg/dose (350 mg) without epinephrine and 500 mg with it. The maximum dose recommended for children over 4 years is 7 mg/kg/dose. Its metabolism is hepatic, and it is classified in Category C in pregnancy.^6^

#### Procaine

Procaine is an ester-type anesthetic whose action starts within 5 min and its duration is short (30–60 min). The maximum dose for adults is 10 mg/kg/dose (350–500 mg) without epinephrine and 14 mg/kg/dose (600 mg) with it. Similar to other anesthetics in this category, its metabolism is plasmatic. It is a Category C in pregnancy (Table 1, Table 2).^1^, ^6^

### Topical anesthetics

Topical anesthetics are available in distinct preparations and vehicles. The mixtures allow compounds to be in liquid state and at higher, although safe, concentrations.^13^

#### Lidocaine

Lidocaine is the most often used anesthetic, either in its isolated form or in association with other components. It belongs to the amide group, which makes this anesthetic less allergenic. It is classified as Category B in pregnancy; however, it is necessary paying close attention to lactating, due to its excretion in breast milk.^8^, ^13^

#### Lidocaine at 4% (Dermomax®)

Lidocaine cream is the most used topical anesthetic worldwide, either alone or in combination with other substances.^8^ It is traded in 4% cream in Brazil. Its systemic absorption changes based on the location and extent of the treated area. Serum peak reaches 0.05–0.16 μg/mL, after the application of 60 g of lidocaine cream in a 400 cm^2^ area. Toxic levels of this substance (>5 μg/mL) lead to cardiovascular and central nervous system disorders. The maximum application area in children weighing up to 10 kg is 100 cm^2^, whereas the maximum application area in children weighing from 10–20 kg is 200 cm^2^. This anesthetic is classified as category B during pregnancy.^8^, ^13^

#### Lidocaine 2.5% + prilocaine 2.5% (EMLA®)

One of the most used topical anesthetics is based on the association between lidocaine 2.5% and prilocaine 2.5%. Systemic absorption depends on the application duration, anatomic location and on the extension of the treated area. Occlusion and prolonged application increase medication penetration. Analgesia reaches 3 mm down in 60 min application and 5 mm down after 120 min. The use of 60 g of medication in 400 cm^2^ of occluded body surface means low toxicity risk. It is allowed to use up to 1 g of anesthetic for, at most, 1 h in newborns (Table 3).^8^, ^13^

**Table 3:** Recommended maximum dose and application área of eutetic mixture of local anesthetics cream (EMLA®).^8^

Age and body weight requirement	Maximum total dose (g)	Maximum application area (cm^2^)	Maximum application time (hours)
0–3 months or <5 kg	1	10	1
3–12 months and >5 kg	2	20	4
1–6 years old and >10 kg	10	100	4
7–12 years old and >20 kg	20	200	4

#### Lidocaine 7% + tetracaine 7% (Pliaglis®)

The lidocaine 7% and tetracaine 7% formation in moisturizers forms a membrane on skin surface and it helps its local absorption. The compound penetrates 6.8 mm down the skin and must be administered 30 min before the procedure. Local reactions include erythema, pallor and edema.^8^

#### Epinephrine

Epinephrine (adrenaline) is a vasoconstrictor often associated with local anesthetics (1–2:100.000). It allows slower systemic reabsorption of the anesthetic by prolonging its effect, reducing its plasmatic peak and promoting hemostasis. This compound must be carefully used in patients treated with beta blockers or in the ones with cardiovascular diseases, peripheral vascular diseases, severe hypertension, pheochromocytoma and hyperthyroidism. However, some studies have shown that its use is safe when it is administered in small doses in patients with stable heart disease.^1^, ^6^, ^14^

Epinephrine addition to local anesthetics also proved to be safe in terminal vascularization sites such as dactyls, hands, feet and genital area.^1^, ^15^

Epinephrine is classified in Category C in pregnancy and its alpha-adrenergic property can cause vasoconstriction in plexus blood from the placenta. However, its use in pregnant women is safe at low doses, since its vasoconstrictor effect limits its systemic absorption and transference to the placenta. Its administration on pregnant women is recommended to be postponed to after childbirth whenever possible.^1^, ^16^

The adopted concentrations range from 1:500.000 to 1:200.000; concentrations 1:100.000 and 1:200.000 are the most often administered ones. These concentrations have similar effect on vasoconstriction, besides prolonging lidocaine duration by approximately 200%.^1^

## Adverse reactions

Local anesthetics are quite safe when they are properly used. However, although rare, they can cause some adverse reactions, including some events with systemic repercussion.

Undesired reactions can be divided into two categories: the ones associated with needle penetration in the skin and the ones associated with the anesthetic solution. Pain, edema, bruise, infection, hyperalgesia and muscular trismus stand out among factors in the first category (needle penetration in the skin).^5^ Reactions associated with the solution concern local or systemic toxicity, allergic and idiosyncratic reactions.^5^

### Local toxicity

Local toxicity is attributed to the straight effects of local anesthetics on the application site, such as pain. It is commonly associated with incorrect techniques either regarding the substance itself or tissue distension in the application site, for instance: bruise, infection, dilacerations of nervous structures and ischemic necrosis.^5^, ^6^

### Systemic toxicity

Local anesthetic systemic toxicity (LAST) is the most severe adverse reaction since it has the potential to kill the patient. It occurs when the plasmatic level in the anesthetic rises to concentrations above the recommended. This reaction can happen suddenly after the application of the anesthetic in the blood stream, or slowly, due to increased serum levels in the anesthetic after the administration of excessive doses, or to reduced medication metabolism.^1^, ^5^, ^17^, ^18^, ^19^

First, patient's present signs of central nervous system activation, which tend to progress: perioral paresthesia, facial paresthesia, disarthria, metallic taste, diplopia, auditory disturbances and seizures. High blood pressure and tachycardia can also be associated with such activation. Symptom progression brings along signs of nervous system depression, which leads to respiratory depression (lidocaine serum concentration higher than 15 μg/mL). The cardiovascular effects come later, and they include myocardial depression, prolonged conduction interval, bradycardia, hypotension and heart failure (lidocaine serum concentration higher than 20 μg/mL).^1^, ^5^, ^17^

Although potentially severe, the systemic toxicity is extremely rare. The necessary dose of local anesthetics for most dermatological procedures is much lower than the dose recommended for each anesthetic – systemic toxicity is associated with high doses of the medication.^1^

Vasques et al. published a literature review, which identified 67 systemic toxicity cases described in 54 articles between 2010 – year when the LAST protocol algorithm was published by the American Society of Regional Anesthesia and Pain Medicine – and 2014. Eight (8) cases, out of the total, emerged after continuous infusion of the local anesthetic, and two other cases happened after the inadvertent administration of the drug in the vessel through venous cannula. The administration technique was adequate for 78% of the 50 patients who received one single dose of local anesthetic. Among these patients, 23% presented systemic toxicity after interscalene block, 16% after epidural block and 13% after penile block. Patients younger than one-year-old were the most affected ones (22%). Seven (7) cases were associated with topical anesthetics (non-injectable), 5 cases were associated with children – 4 of these children were younger than 4 years. Systemic toxicity was only observed in two dermatological patients: two children evolved to methemoglobinemia after EMLA administration.^17^

The prospective study by Starling et al. showed that in-office dermatological procedures recorded very low complication rates. After ten years studying the states of Florida and Alabama (U.S.A.), they did not identify any complication related to local anesthetics in dermatological procedures.^2^

The longitudinal study by Alam et al. included a research with 437 dermatological surgeons in the United States and it also highlighted intoxication cases caused by lidocaine use during surgical procedures conducted for ten consecutive days by the doctors. The maximum dose of the medication was 6.54 mL in excisions and 15.85 mL in reconstructions. The incidence of adverse reactions related to local anesthesia was 0.15% – reactions were moderate in 0.13% of the cases (dizziness, drowsiness and tachycardia caused by epinephrine).^20^

A series of 20,021 patients was assessed by Barrington and Kluger in a multicenter study conducted in Australia and New Zealand between January 2007 and May 2012. Patients were subjected to 25,336 blocks of peripheral nerves with and without ultrasonography. Patients younger than 13 years old were excluded from the experiment. There were 22 episodes of systemic toxicity, which resulted in the incidence 0.87 per 1000 blocks. Only one patient evolved to heart failure after the intravenous injection of the anesthetic during a paravertebral block procedure. The injection site, the used anesthetic type, the dose per weight and patient body mass were the predictor factors for the incidence of complications.^21^

Sites et al. did not identify any heart failure case in a prospective study with 12,668 patients subjected to peripheral regional anesthesia from July 2003 to February 2011 in a hospital in the United States. Only one systemic toxicity case was recorded (incidence 0.08 per 1000).^22^

The treatment of systemic toxicity consists in stopping anesthetic administration and requesting immediate medical assistance. Then, basic and advanced life support procedures must be initiated: preservation of airways and of the cardiovascular system. The specific treatment in heart failure cases counts on 20% lipid emulsion therapy.^5^, ^6^

### Hypersensitivity reactions

#### Type 1 hypersensitivity reactions (IgE-mediated reactions and anaphylaxis)

The prevalence of true IgE-mediated allergy to local anesthetics is estimated to be lower than 1%.^6^ The literature review by Bhole and collaborators published in 2012 assessed 23 series of cases that included 2978 patients between 1950 and 2011. Only 23 of these patients presented true type 1 hypersensitivity reaction to topical anesthetics.^23^

Local reactions are more common and include urticaria and angioedema without respiratory compromise. The treatment consists in administering antihistamines and closely observing patients.^24^

Immediate systemic hypersensitivity reaction (anaphylaxis) symptoms are observed in the first 30 min after the exposure to the anesthetic. These symptoms must be identified immediately by the doctors. Overall, two or more systems are involved or there are evidences of respiratory or cardiovascular compromise. Symptoms include dyspnea, cough, wheezing, hypotension and tachycardia. The treatment must start with the immediate intravenous administration of vasoconstrictors (epinephrine).^24^

In case of allergic reaction, one can use ester-type anesthetics due to the low crossed reaction between them, as well as anesthetics belonging to the amide group. Other options would be the administration of diphenhydramine or bacteriostatic saline solution (0.9% benzyl alcohol saline solution) in biopsies or small skin excisions.^1^

#### Type IV hypersensitivity reactions

The late hypersensitivity reaction includes allergic contact dermatitis. According to the retrospective study by To et al., who assessed 1819 patients subjected to patch test in Canada, its incidence reaches 2.4%. Benzocaine is the most common allergen (45%), which is followed by lidocaine (32%) and dibucaine (23%).^25^ It is estimated that its incidence reaches 3.4% in the United States.^26^

The clinical frame 24 and 48 h after the exposure to the agent is featured by erythema, edema, peeling, infiltration, blistering and skin crusting. The symptoms include burning and itching.^27^

Patients who present patch test positive for lidocaine must be subjected to intradermal injection of 0.1% lidocaine to confirm allergic contact dermatitis due to the substance. Other local anesthetics must be also tested.^25^

#### Vasovagal reaction

This reaction results from anxiety and from patients’ sense of pain and fear of the needle or of the procedure itself. It stimulates the parasympathetic system and its symptoms can be similar to allergic reaction: dizziness, sweating, nausea, bradycardia and hypotension. In extreme cases, there can be syncope. The treatment consists in calming the patient down, putting him/her in Trendelenburg position and in applying cold compresses on the forehead.^24^

### Topical anesthetics

Local reactions to topical anesthetics include erythema, pallor and edema. Anesthetic creams should not be directly applied to the eyes, oral mucosa and internal auditory canal due to the risk of triggering local irritation. In addition, it is imperative being careful about the amount of medication used at the time to apply it to the genital mucosa.^8^

Systemic reaction, such as methemoglobinemia, central nervous system dysfunctions and cardiotoxicity, although rare, can happen.

Methemoglobinemia is a particular concern for the pediatric population, because of the immature metabolism of methemoglobin in children under 3 month-old.^6^ This condition can be triggered by EMLA® use, since prilocaine has the potential to prevent oxygen transport by hemoglobina.^8^ Methemoglobin levels from 15% to 30% result in cyanosis. Levels from 30% and 50% result in dyspnea, tachycardia and headache. Levels higher than 50% cause lethargy and coma.^8^

The literature review by Tran and Koo (2014) includes 12 studies about the safety of the topical anesthetic EMLA. Twelve (12) LAST cases were identified and 9 of them were observed in children.^28^

The 7% lidocaine 7% tetracaine-based cream can cause moderate and transient erythema and pallor at the application site.^8^

It is important observing that some techniques can increase the dermal absorption of topical anesthetics by the corneal layer of the skin, such as the ablative laser.^29^ Product application on inflamed surfaces, or on large areas of body surface, can also increase the risk of absorption and toxicity.^8^

The medication must be immediately washed off the skin surface if any sign of toxicity is identified. The patient must be put in supine position and his/her vital signs must be assessed. The specific treatment must start based on the signs and symptoms.^8^

## Application techniques

The correct anesthetic application technique assures procedure safety and comfort. The doctor must wear gloves and make proper skin antisepsis. The site to be treated must be marked prior to the administration of the anesthetic in order to avoid local distortion due to the volume of injected medication.^5^

Thin and small needles must be used whenever possible, because they enable less discomfort. Slow infusion, skin vibration, use of warm solution, skin cooling and minimal injections in the skin also help achieving comfort sensation. In some cases, topical anesthetics are recommended to be applied to the skin prior to the injectable anesthetic, mainly in children.^1^, ^5^

Topical anesthetic must be applied to intact skin, i.e., on skin free from erosion or eczema. It is important avoiding the contact between the medication and the ocular mucosa, as well as the use of anesthetics belonging to the amide group in patients with kidney issues, and the use of EMLA anesthetics in newborns.^8^

## Safety of anesthetics

According to the American guidelines published in 2016, local anesthetics are safe medications to be used in in-office dermatological procedures. Systemic toxicity episodes and episodes of anaphylactic reaction to local anesthetic are rare and many authors recommend them for surgical procedures conducted outside the hospital environment.^1^, ^2^, ^30^, ^31^, ^32^

The prospective study by Starling et al. (2012) collected data about complications in outpatient procedures in Florida State from 2000 to 2010, and in Alabama State from 2003 to 2009, both in the United States. In Florida, they reported 46 deaths and 263 complications during surgical procedures: 45% of these complications happened during procedures conducted by plastic surgeons. Only four complications happened during procedures conducted by dermatologists, without deaths (1.3% of the cases): one episode of vasovagal reaction after liposuction in patient subjected to general anesthesia; one case of short-duration atrial fibrillation in patient subjected to skin excision; one incorrect surgical excision in Mohs surgery; and one episode of second degree burn in bedridden patient using home oxygen, who was sedated for the dermatological procedure. In Alabama State, they reported 3 deaths and 49 complications during outpatient procedures. Plastic surgery was the medical specialty recording the highest index of complications (42.3% of the cases). Only one complication happened during procedures conducted by dermatologist, and it did not evolve to death; this number corresponds to only 1.3% of the cases: only one patient presented infection caused by methicillin-resistant *Staphylococcus aureus* after a melanoma excision. There was no complication after cosmetic procedures based on local anesthesia performed by dermatologists.^2^

The research by Hanke, Bernstein and Bullock included 15,336 patients subjected to liposuction with tumescent anesthesia conducted by dermatological surgeons; their study showed complications in only 0.38% of the cases. Two patients had cardiac arrhythmia and two presented persistent tachycardia. No death was reported.^33^ Klein and Jeske also showed safe lidocaine serum levels when they used high doses of tumescent anesthesia in patients subjected to liposuction.^34^

The prospective cohort study by Alam et al. recorded similar results for 19 patients subjected to micrographic Mohs surgery. These authors measured six lidocaine serum levels administered in three different times during a surgical procedure. There was no lidocaine serum level increase at toxic doses, even during procedures that have used higher doses of the anesthetic: the highest lidocaine serum level found by them was 0.3 μg/mL.^35^

In 2012, Walsh and collaborators published a study showing that dermatologists’ knowledge about the doses and toxicity of local anesthetics is satisfactory. However, the treatment applied to systemic toxicity with lipid emulsion was properly informed by 21.7% of the participants in the study, and this index was considered low by the authors.^36^

The retrospective study by Kvisselgaard and collaborators ruled out hypersensitivity reaction in all 164 patients in their study, who were previously referred to an Allergology and Immunology clinic with the suspicion of type 1 reaction to local anesthetics between 2010 and 2014. These authors believe that this event was rare due to the use of these medications.^37^

Although adverse reactions to anesthetics are not frequent, doctors must be careful during their use. The selection of the adequate medication, the identification of risk signs and the handing of complications are essential knowledge for dermatologists.^6^

## Special situations

### Pregnancy and lactation

Lidocaine is classified in Category B in pregnancy and epinephrine in Category C.^1^, ^16^ Doctors must be careful in the use of anesthetics in this category of patients due to their increased local sensitivity and to their systemic absorption of these medications in this phase. It is recommended to avoid intravenous injection because of the risk for maternal and fetal cardiotoxicity.^38^

### Pediatric and elderly populations

The application of local anesthetics in the pediatric population demands caution. It is important differentiating signs of fear from effects on the central nervous system.^6^ Mucocutaneous absorption in newborns is higher and faster than in adults; besides, the link between anesthetics and plasma proteins is fragile in the pediatric population, which results in high intoxication.^39^

Local anesthetic clearance in elderly is lower due to organic dysfunction and compromised circulation. Moreover, neural changes make these patients more sensitive to these medications. In many cases, lower doses are necessary in order to accomplish the same analgesia.^6^, ^40^

### Comorbidities

Kidney dysfunction can affect the systemic circulation of local anesthetics. Kidney issues and uremia can increase the local absorption of these medications and reduce ropivacaine and bupivacaine clearance.^6^, ^38^

Altered blood circulation reduces the metabolism of substances in the liver and kidneys, thus leading to reduced clearance of anesthetics. Heart failures reduce the local absorption of these medications due to low tissue perfusion. However, increased anesthetic concentrations can occur in the central nervous system. Epinephrine must be used with caution and avoided in some cases.^6^, ^38^

## Final considerations

Local anesthetics can be considered safe medications for use by dermatologists. Although some adverse reactions are considered severe, such as systemic toxicity and anaphylactic reaction, their occurrence is rare. Proper management of these medications, adequate application technique and knowledge about adverse events, and their specific treatment, reduce risks associated with local anesthetics and make their in-office application viable.

On September 18, 2017, new rules for operation in private medical offices, outpatient and hospitals came into force in Brazil. They were published by the Federal Council of Medicine (Conselho Federal de Medicina – CFM) after some changes in Resolution CFM n° 2.056/2013.

Offices or services that perform invasive medical procedures involving the risk for anaphylaxis, and respiratory and cardiovascular failure – including places where only local anesthetics without sedation are used – were kept in Group 3 Dermatology Offices. In this case, besides the basic structure for propaedeutic, these offices and services must have input and equipment for the therapeutics and treatment of anaphylactic reactions, and for immediate relief of complications caused by therapeutic intervention.

According to evidences shown in the present study about the safety profile of local anesthetics for dermatological procedures, the authors suggest a review about the current demands of the resolution in force. Dermatological procedures such as the excision of small cutaneous lesions, which demand safe doses of local anesthetics (<6.4 mL of lidocaine)^20^ and are conducted by clinical dermatologists on a daily basis, could be reallocated for level 2 procedures.

## Financial support

None declared.

## Author's contribution

Ana Carolina Figueiredo Pereira Cherobin: Conception and planning of the study; elaboration and writing of the manuscript; collection, analysis, and interpretation of data; critical review of the literature; critical review of the manuscript.

Glaysson Tassara Tavares: Approval of the final version of the manuscript; conception and planning of the study; effective participation in research orientation; critical review of the manuscript.

## Conflicts of interest

None declared.
